# Translation, transcultural adaptation, and convergent validity of the Arabic version of the Mukbang addiction scale

**DOI:** 10.1186/s40337-024-01036-6

**Published:** 2024-07-04

**Authors:** Wizra Saeed, Nisma Merdad, Rizwana Amin, Tabassum Rashid, Souheil Hallit, Feten Fekih-Romdhane

**Affiliations:** 1 Psychology Department, College of Humanities, Effat University, Jeddah, 21478 Saudi Arabia; 2 School of Medicine and Medical Sciences, Holy Spirit University of Kaslik, P.O. Box 446, Jounieh, Lebanon; 3 Applied Science Research Center, Applied Science Private University, Amman, Jordan; 4 The Tunisian Center of Early Intervention in Psychosis, Department of Psychiatry “Ibn Omrane”, Razi Hospital, Tunis, Manouba 2010 Tunisia; 5 Faculty of Medicine of Tunis, Tunis El Manar University, Tunis, Tunisia

**Keywords:** MAS, Mukbang addiction, Addictive mukbang watching, Arabic, Psychometric properties

## Abstract

**Introduction:**

The present study evaluated the psychometric properties of the Arabic translation of the Mukbang Addiction Scale (MAS) among Arabic-speaking adults from the general population. Specifically, it aimed to assess the factorial structure through a confirmatory factor analysis, determine the composite reliability through Cronbach alpha and McDonald’s omega scores, assess gender invariance, and evaluate the convergent validity by examining its correlation to eating addiction and psychological distress.

**Methods:**

A total of 370 individuals with a mean age of 21.94 ± 2.29 years participated in this study, which was conducted using an online platform. The participants were surveyed on demographic information, mukbang addiction, food addiction, and psychological distress. Translation was conducted using the forward and backward technique.

**Results:**

The findings demonstrated excellent internal consistency of the Arabic MAS (McDonald’s omega coefficient = 0.93). Confirmatory factor analyses validated the one-factor structure of the scale, while establishing measurement invariance across sex at the configural, metric, and scalar levels. No sex differences were observed in the Mukbang addiction levels. Lastly, the MAS scores were significantly and positively correlated with food addiction and psychological distress, supporting their convergent validity.

**Conclusion:**

The current research provides evidence supporting the reliability and validity of the Arabic version of the MAS as a self-report method for assessing addictive Mukbang watching. While further validations are needed to corroborate the present findings, this measure can be effectively utilized across different fields, including schools, mental health centers, and researchers aiming to understand this global phenomenon.

**Plain English Summary:**

Mukbang refers to individuals or hosts consuming large quantities of food while interacting with their audiences through recorded video or a live stream. The Mukbang phenomenon has gained substantial popularity among young individuals over the past years, rising concerns about its potential impact on their eating habits and health, especially when overconsumed. Overconsumption of mukbang content, or Mukbang addiction, aligns with the core features of addiction, including compulsive engagement, tolerance, and withdrawal symptoms, making it comparable to other addictive behaviors. Mukbang addiction can lead to detrimental effects on mental and physical health, including neglect of balanced nutrition, disordered eating habits, obesity, feelings of guilt, shame, poor self-esteem, distorted self-images, body dissatisfaction, heightened anxiety or depression, and social isolation. Given these significant impacts, valid and reliable tools are crucial to enable an accurate assessment of Mukbang addiction. This study proposes to translate, adapt and examine the psychometric properties of the Mukbang Addiction Scale (MAS) in a sample of Arabic-speaking individuals from the general population. Findings showed that the six MAS items loaded on a single factor with strong internal consistency and good convergent validity, preliminarily indicating its potential validity and reliability for assessing addictive mukbang-watching among Arabic-speaking individuals.

## Introduction

The term “mukbang” comes from the Korean words “muk-da” (meaning “to eat”) and “bang-song” (meaning “broadcast”). Mukbang phenomenon refers to individuals or hosts consuming large quantities of food while interacting with their audiences via a live stream or recorded video [1]. As a result of the wide variety of food presented in a visually stimulating manner in mukbang videos, mukbang videos have gained substantial popularity among young adults; watching these videos often triggers a sense of pleasure that is similar to enjoying a satisfying meal [2–4].

One reason behind the rising popularity of mukbang among adolescents is its unique appeal as a form of entertainment, escapism, and its ability to foster a sense of community. Through online platforms, adolescents can connect with like-minded individuals who share their passion for food and eating challenges [5, 6]. However, the rising popularity of mukbang raises concerns about its potential impact on adolescent eating habits and health. The key area of concern is the prospective influence mukbang may have on unhealthy eating behaviors [7]. As adolescents observe excessive food consumption during these videos, they might be more likely to adopt similar habits in their daily lives. This could lead to overeating or indulging in unhealthy foods high in calories, sugar, and fat [8]. Consequently, the prevalence of obesity and other diet-related health issues among adolescents could increase. Moreover, mukbang’s focus on quantity rather than quality may lead to a neglect of balanced nutrition [9]. This is especially essential as obesity in children is a severe problem in the Arab world [10]. In fact, problematic mukbang viewing was related to disordered eating habits such as purging and binge eating [11–13].

According to DSM-5-TR, addiction is described as a persistent involvement in pleasurable activities, even when there are negative outcomes involved [14]; within the realm of mukbang, individuals may find themselves persistently viewing these videos despite adverse impacts on their mental and physical well-being, such as excessive eating or the formation of bad eating patterns and psychological anguish Compulsion to watch mukbang videos as a coping strategy could lead to compulsive behaviors similar to addiction [12]. The biological phenomenon in the brain happens as food displays and eating sounds trigger the release of dopamine in the brain. The dopamine rush causes viewers to seek satisfaction and pleasure repeatedly, leading to an addictive cycle [15]. Several critical research studies examined the potential negative psychological effects of mukbang watching and shed light on their potential adverse effects. Overconsumption of mukbang content has been shown to negatively affect mental well-being by creating feelings of guilt, shame, poor self-esteem, distorted self-images, body dissatisfaction, heightened anxiety or depression, and social isolation due to users’ preference for online social engagement over in-person ones [11, 12, 16–18].

Furthermore, this addictive cycle is accountable for the development of tolerance, by the growth in the number of mukbang video viewings required to achieve the desired impact of fulfillment and pleasure. Moreover, addiction is characterized by withdrawal symptoms that arise when the addictive stimulus is taken away. Individuals who frequently watch mukbang videos may experience feelings of restlessness, irritability, or cravings when they are unable to access these videos, indicating dependence on the stimulus. Overall, the addictive nature of mukbang aligns with the core features of addiction, including compulsive engagement, tolerance, and withdrawal symptoms, making it comparable to other addictive behaviors [2, 7, 19, 20]. Although online addiction encompasses a diverse range of behaviors, such as excessive gaming, social media usage, and gambling, it is essential to focus on mukbang as a specific subtype of online addiction. Both mukbang and other manifestations of internet addiction exhibit fundamental characteristics of addiction, including a lack of control, obsession, and negative outcomes.

There are more generally additional critiques raised when mukbang is taken into account with other forms of internet addiction. Mukbang addiction, like different types of online addiction, is typically marked by compulsive behavior, lack of self-control, and adverse outcomes. Nevertheless, critics contend that directing attention towards particular online activities, such as watching mukbang videos, could potentially contribute to problematic internet usage. This, in turn, may have adverse effects on mental well-being, manifesting as feelings of guilt, shame, low self-esteem, distorted self-perception, dissatisfaction with one’s body, increased anxiety or depression, and social isolation resulting from a preference for online social interaction over face-to-face interactions [21–23]. A recent research study published in the Body Image journal examined the correlation between content related to making and individuals’ body image and eating behaviors. The study revealed that there is a positive correlation between watching mukbang videos and increased levels of body dissatisfaction and unhealthy eating behaviors. This raises the possibility that consuming mukbang content may have detrimental psychological consequences [24, 25]. 

The Compensatory Internet Use Theory and the I-PACE (Interaction of Person-Affect-Cognition-Execution) model are two well-known conceptual frameworks in the study of internet addiction. The I-PACE model proposes that internet addiction arises from the interplay of individual traits (Person), emotional states (Affect), mental processes (Cognition), and the capacity to control one’s actions (Execution). This concept suggests that certain inherent characteristics, such as individual personality traits and emotional states, might give rise to cognitive biases and impairments. These impairments, in turn, have a significant impact on behavior and the probability of developing internet addiction. Conversely, the Compensatory Internet Use Theory suggests that individuals may excessively utilize the internet as a means to compensate for unfulfilled requirements in their offline lives, such as lacking social connections or meaningful activities. Both theories emphasize the intricate interaction of psychological, emotional, and cognitive elements in the formation and perpetuation of internet addiction, offering significant perspectives for therapeutic and preventative approaches [26–30].

The Mukbang Addiction Scale (MAS) was developed in 2021 to assess Mukbang addiction behaviors in Turkey [7]. The scale was based on the Bergen Facebook Addiction Scale and modified to replace the word *Facebook* with the word *mukbang watching* [31]. It consists of six items which assess different aspects of mukbang addiction such as the frequency and duration of viewing mukbang videos, cravings that result from viewing the videos, and any negative consequences. The items are scored on a 5-point Likert scale ranging from “very rarely” to “very often”. For example, “*How often in the past year have you spent a lot of time thinking about mukbang or planned watching mukbang*?”. The Arabic version of this scale has not yet been evaluated.

The Arabic version of the MAS holds significant importance due to its extensive reach among over 100 million people worldwide. Arabic, being widely spoken, makes it one of the most prevalent languages worldwide. By translating the MAS into Arabic, a wider audience will be able to access and benefit from it. Developing a standardized Arabic scale will enhance researchers and mental health providers’ comprehension of the phenomenon among Arab societies. Given the significant impact of cultural and linguistic elements on the perception and treatment of addiction, it is crucial to have a culturally tailored scale to ensure effective therapies [32].

Moreover, conventional beliefs that encourage overeating as a sign of prosperity and generosity may contribute to the acceptance of mukbang among Arab individuals. As mukbang addiction potentially affects mental and physical health, understanding the prevalence and severity of mukbang addiction among Arab speaking people is crucial for developing effective prevention and intervention strategies [23, 33]. Additionally, providing a valid measure of mukbang addiction for use among Arabs may enable investigation of the psychological and social consequences of this addictive behavior, and shed light on its impact on mental health, eating habits, body image perception, academic performance, and overall well-being [34]. In this context, this study aimed to assess the validity and reliability of the Arabic version of the MAS in adults from the Arabic-speaking community of Saudi Arabia. It was hypothesized that (1) the Arabic version of MAS would replicate the one-factor structure proposed in the original version and (2) the scale would show acceptable internal consistency and invariance across sex groups, (3) MAS scores would correlate with measures of food addiction and psychological distress, supporting convergent validity.

## Methods

### Participants

The present study employed an online questionnaire and convenience sampling technique to approach the participants. An online post was shared on social media platforms (such as WhatsApp, Instagram, and Facebook), and consenting participants were able to fill out the questionnaire. The content of the post included the information and consent form, as well as the questionnaire. All participants in the current study were Arabic-speaking adults aged 18 years or older who were originating from, and residing in in Saudi Arabia at the time of the survey. Anyone under the age of 18 or residing outside of Saudi Arabia was excluded from the study. A total of 370 adults with a mean age of 21.94 ± 2.29 years participated.

### Procedures

A Google Form consisted of consent sheet, demographic information, MAS, Yale Food Addiction Scale and the Depression Anxiety and Stress Scale (DASS-8) was prepared and shared on social media sites. Participation in the study was voluntarily, and young adults willing to participate filled out the questionnaire. The research team has continued to share the questionnaire on social media sites for a 6-month time period. The research data supporting the findings has been securely stored on the server of a Saudi university for maintaining its integrity for a period of seven years following its publication.

### Adaptation and translation

Permission to translate and validate the MAS was obtained from Dr. Kagan Kircaburun. For this study, the MAS was translated using the ‘forward-backward-forward’ technique aligned with the Principles of Good Practice for Translation and Cultural Adaptation [35]. First, the questionnaire was prepared by the researchers. In order to address the research aims effectively, the researchers selected the appropriate scales to be included in the questionnaire (preparation). Initially, the questionnaire was translated into Arabic by a specialized translator and two experts in the field. The translator collaborated with two experts who had a deep understanding of both the Arabic and English versions of the questionnaire (forward translation and reconciliation). Subsequently, the translated version was converted back into English by another translator (back translation) ensuring that it aligns with the original questionnaire in terms of content, tone, and wording. Both experts and the researchers actively participated in the process of matching and evaluating the theoretical equivalence (harmonization). This involved comparing the translated versions with the original questionnaire, identifying any discrepancies or inconsistencies, and making necessary adjustments. To establish the final version in Arabic, the researchers and experts actively participated in the process (proofreading and finalizing the document). The research team carefully reviewed all aspects of the questionnaire, including grammar, syntax, and overall coherence.

### Measures

Demographic variables included age, sex and education level, in addition to the following scales:

#### The modified version of yale food addiction scale

The original Yale Food Addiction Scale, which was developed to assess signs of addictive-like eating behavior, was introduced by Gearhardt, Corbin, and Brownell in 2009 [36]. The scale was later modified to include only nine items and this modified scale was validated in Arabic in Lebanon [37, 38]. The scale consists of nine questions that are aligned with seven DSM-IV-TR dependence criteria with items such as criteria such as “*I ate to the point I felt physically ill*”. Each question receives a score on a Likert scale ranging from 0 (never) to 4 (four times a week to daily). The internal consistency was found to be acceptable, with Cronbach’s alpha and McDonald’s omega exceeding the acceptable threshold of 0.7 (ω = 0.89; α = 0.89).

### The Depression, anxiety, stress scales (DASS-8)

The DASS was first developed by Lovibond and Lovibond in 1995 to assess for symptoms of depression, anxiety and tension/stress [39]. A shortened version was later created and validated in an Arabic sample [40, 41]. It is comprised of eight items, rated from 0 (does not apply to me) to 3 (applies to me a lot or most of the times), with items such as “*I felt that I had nothing to look forward to”.* The internal consistency was found to be acceptable, with Cronbach’s alpha and McDonald’s omega exceeding the acceptable threshold of 0.7(ω = .90; α = .90).

### Statistical analysis

The dataset did not contain any missing responses. In order to determine the factor structure of the MAS, we conducted a Confirmatory Factor Analysis (CFA) with the data collected from the total sample, using SPSS AMOS v.28 software. Considering a sample size of three to twenty times the number of variables in the scale, 120 participants were required [42]. In this study, we intended to test the original scale model. The maximum likelihood method was used to estimate the parameters. Calculated fit indices were the normed model chi-square (χ²/df), the Steiger-Lind root mean square error of approximation (RMSEA), the Tucker-Lewis Index (TLI) and the comparative fit index (CFI). Values ≤ 5 for χ²/df, and ≤ 0.08 for RMSEA, and 0.95 for CFI and TLI indicate good fit of the model to the data [43]. In order to verify convergent validity, the Average Variance Extraction (AVE) was calculated (values of 0.5 or more are considered adequate). At first, multivariate normality was not verified (Critical ratio > 5; Bollen-Stine *p* = .002); therefore, nonparametric bootstrapping was performed. A value of greater than 0.70 indicates adequate composite reliability in both subsamples using McDonald’s ω and Cronbach’s alpha.

Multi-group CFA was conducted to examine sex invariance of MAS scores [44] using the total sample. The measurement invariance was assessed at three levels: configural, metric, and scalar [45]. We accepted ΔCFI ≤ 0.010 and ΔRMSEA ≤ 0.015 or ΔSRMR ≤ 0.010 as evidence of invariance [44].

The SPSS software v.25 was used for the remaining statistical analysis. The MAS score was considered normally distributed since the skewness and kurtosis varied between − 1.96 and + 1.96. The Student’s t-test was used to compare two means, whereas the Pearson test was used to correlate two continuous variables. *P* < .05 was deemed statistically significant.

## Results

Participants’ characteristics are displayed in Table 1. The majority of individuals were females (82.2%) and had a university educational level (88.4%).

**Table 1:** Characteristics of the sample (*n* = 370)

Sex	
Male	66 (17.8%)
Female	304 (82.2%)
Educational level	
Secondary or less	43 (11.6%)
University	327 (88.4%)
Age (years)	21.94 ± 2.29
Mukbang addiction	11.11 ± 5.93
Food addiction	12.20 ± 7.91
Psychological distress	11.48 ± 6.21

### Confirmatory factor analysis of the MAS scale

**Fig. 1:**
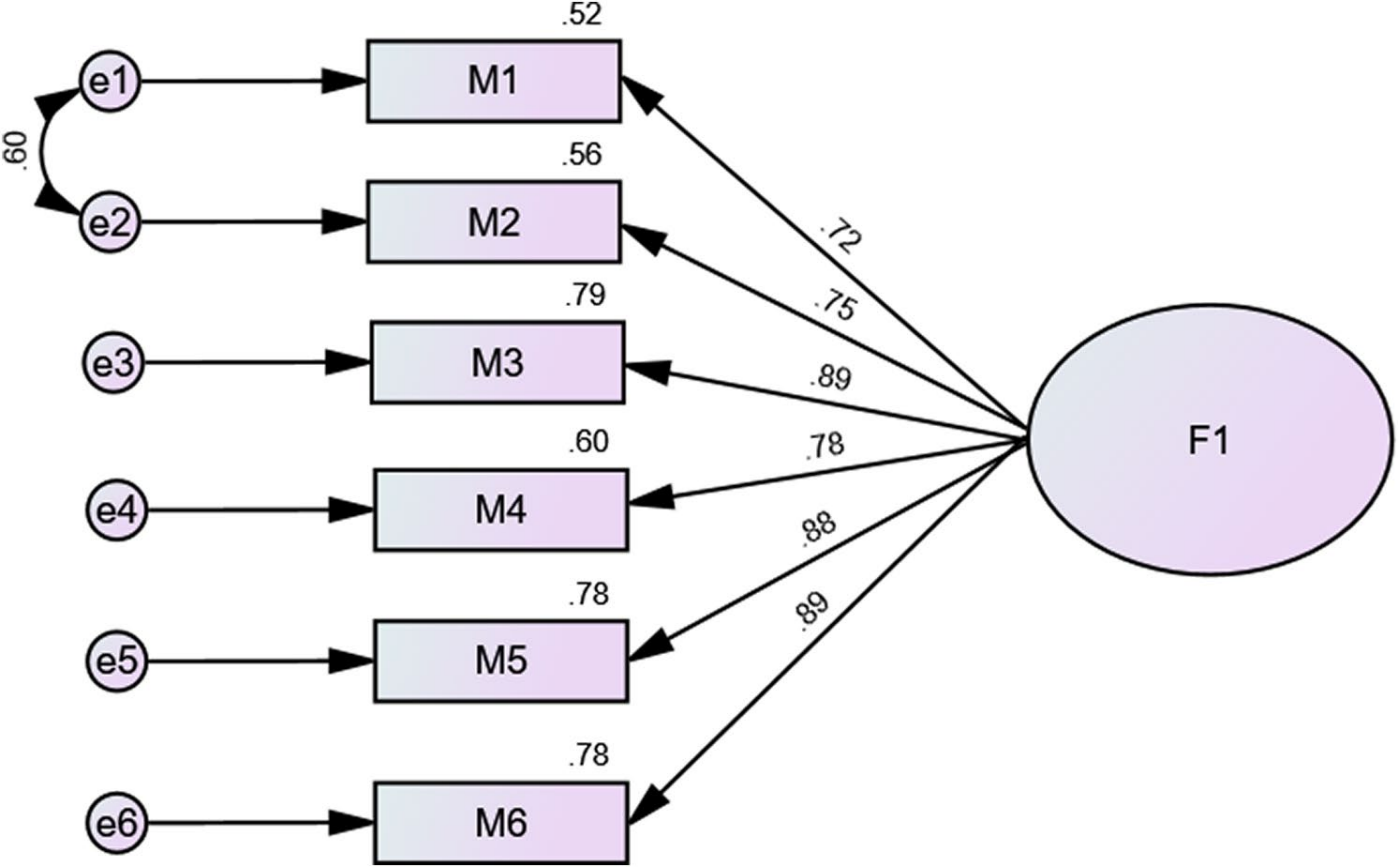
Standardized estimates of factor loadings from the confirmatory factor analysis (CFA) in the total sample

CFA indicated that fit of the one-factor model of MAS scores was poor: χ^2^/df = 173.46/9 = 19.27, RMSEA = 0.223 (90% CI 0.194, 0.252), SRMR = 0.054, CFI = 0.908, TLI = 0.847. We added a correlation between residuals of items 1 and 2 since the modification index was high; the numbers improved as follows: χ^2^/df = 32.49/8 = 4.06, RMSEA = 0.091 (90% CI 0.060, 0.125), SRMR = 0.024, CFI = 0.986, TLI = 0.974. The standardized estimates of factor loadings were all adequate (see Fig. 1). Internal reliability was excellent (ω = 0.93; α = 0.93). The AVE value was satisfactory = 0.67.

## Sex invariance

Indices suggested that configural, metric, and scalar invariance was supported across sex (Table 2). No significant difference was found between males (*M* = 11.56, *SD* = 6.83) and females (*M* = 11.01, *SD* = 5.72) in terms of Mukbang behaviors, *t*(368) = 0.69, *p* = .492.

**Table 2:** Measurement Invariance across sex in the total sample

Model	CFI	RMSEA	SRMR	Model Comparison	ΔCFI	ΔRMSEA	ΔSRMR
Configural	0.972	0.096	0.020				
Metric	0.969	0.087	0.022	Configural vs. metric	0.003	0.009	0.002
Scalar	0.959	0.091	0.023	Metric vs. scalar	0.010	0.004	0.001

Note CFI = Comparative fit index; RMSEA = Steiger-Lind root mean square error of approximation; SRMR = Standardized root mean square residual

### Concurrent validity

Higher Mukbang addiction scores were significantly associated with more psychological distress (*r* = .29; *p* < .001) and more food addiction (*r* = .26; *p* < .001).

## Discussion

This study aimed to evaluate the psychometric properties of the MAS in Arabic-speaking individuals residing in Saudi Arabia. As anticipated, the Arabic version of the MAS demonstrated strong internal consistency and convergent validity. It also showed that the one-factor model proposed by the original developers of the scale was fitting. The study supports the reliability and validity of the Arabic version of the MAS.

Using the one-factor model proposed by the original scale developers, a CFA was performed to examine fit indices in the current study [46]. According to the results, the one factor model fits the data well only after adding a correlation between residuals of items 1 and 2. The factor loadings were appropriate ranging from 0.78 to 0.52 [47]. Furthermore, the reliability of the scale exhibited excellent internal consistency, with a Cronbach alpha score of 0.93, mirroring the internal reliability of the original article, which stood at 0.95 [46]. In addition, this study assessed the McDonald’s ω scores (ω = 0.93) which was not assessed in the previous validation studies for this scale. These results demonstrate that the MAS has good internal consistency. The results also demonstrated sex invariance within the sample. This indicates that the scale exhibits equal applicability in Arabic-speaking men and women participants. In this study, there was no significant sex difference in mukbang behaviors. This contrasts with another study that indicates that men had lower MAS scores than women [23]. However, it is important to note that this study utilized a heavy female sample, which may have impacted the results.

The final finding of the study provided support for concurrent validity, by establishing a correlation between mukbang addiction and some psychological problems, specifically, psychological distress and food addiction. This finding is congruent with a previous study that reported a link between problematic mukbang watching and psychological distress [23]. There are several plausible explanations for this phenomenon. One possibility is that individuals may engage in mukbang viewing as a means of escaping from daily stressors [48, 49]. In terms of food addiction, recent research has shed light on the association between mukbang addiction and various purging and binge eating behaviors, as well as disordered eating habits [7, 11, 12]. The research conducted in this field indicates that individuals who exhibit addictive behavior towards mukbang may utilize watching these videos as a strategy to prevent the occurrence of binge eating episodes. The reasoning behind this phenomenon could be that individuals experience a sense of “having someone eat for them” while viewing mukbang videos, which effectively decreases the likelihood of engaging in binge eating habits [12]. This could suggest that individuals with a tendency to exhibit addictive behaviors towards food may exhibit a greater inclination towards watching mukbang. This result aligns with previous research that demonstrated a correlation between watching mukbang videos and engaging in unhealthy eating habits [24, 25]. Additionally, it is plausible that the act of watching mukbang may exacerbate the underlying feelings of food addiction in these individuals. Overall, this is consistent with other research that suggested that the addictive nature of mukbang exhibits similarities with the core features of addiction [2, 7, 19, 20]. In addition, one noteworthy observation derived from a study analyzing comments posted beneath mukbang videos on YouTube was the high percentage of comments reflecting a history of binge eating [12]. Furthermore, these comments frequently emphasized how viewing mukbang videos could set off a resurgence of uncontrollable eating episodes. These discoveries raise concerns about the possibility that mukbang may act as a trigger for individuals who have a history of binge eating. Overall, these findings merit heightened attention.

### Study limitations

This study showed encouraging results when evaluating the reliability and validity of the MAS in a sample of Arabic-speaking adults in Saudi Arabia. However, it is essential to acknowledge its limitations. Firstly, it is important to note that the sample was from a single Arab country, which may limit the generalizability of the findings to the rest of the country or the Arab world. Therefore, it is crucial to replicate this study in more representative samples from other Arabic-speaking countries or communities. Secondly, this research utilized a cross-sectional design, which precluded the examination of certain psychometric properties, such as test-retest reliability. Another limitation of this study is that the participants self-reported their answers, which raises the possibility of social desirability bias. This refers to individuals providing information in a manner that aligns with societal norms or expectations rather than their true experiences and behaviors. Additionally, it is important to acknowledge that our sample relied on convenience sampling which was disproportionately composed of highly educated females. This may not represent the broader population and may have affected the results obtained. The scale still needs to be validated in clinical samples (e.g., individuals with eating disorders). Finally, convergent validity of the MAS using another measure of the same construct (e.g., the Problematic Mukbang Watching Scale [50]) was not performed in the context of the present study because of the unavailability of such measures in the Arabic language. At the same time, it is of note that there is no gold standard assessment of the Mukbang Phenomenon to compare to.

## Conclusion

Pending future studies addressing these limitations, the Arabic MAS showed satisfactory psychometric properties, preliminarily indicating its potential reliability for assessing addictive mukbang-watching. As the popularity of mukbang-watching continues to grow worldwide, including in the Arab world, and as a link between this phenomenon and disordered eating habits and psychological issues has emerged, this scale can be employed by clinicians, mental health professionals, and researchers to gain deeper insights into this phenomenon. By highlighting the validity and reliability of this Arabic MAS, future research may be conducted, ultimately enabling the implementation of evidence-based treatment and support programs in Arab settings.

## Data Availability

The datasets generated and/or analyzed during the current study are not publicly available but are available from the corresponding author on reasonable request.
